# Incidence and Survival Changes in Patients with Esophageal Adenocarcinoma during 1984–2013

**DOI:** 10.1155/2019/7431850

**Published:** 2019-12-12

**Authors:** Zhang Haiyu, Pei Xiaofeng, Mo Xiangqiong, Qiu Junlan, Zheng Xiaobin, Wang Shuncong, Sun Huanhuan, Ma Haiqing

**Affiliations:** ^1^Department of Oncology, The Fifth Affiliated Hospital of Sun Yat-sen University, Zhuhai, Guangdong 519000, China; ^2^Department of Thoracic Oncology, The Fifth Affiliated Hospital of Sun Yat-sen University, Zhuhai, Guangdong 519000, China; ^3^Department of Gastrointestinal Surgery, The Fifth Affiliated Hospital of Sun Yat-sen University, Zhuhai, Guangdong 519000, China; ^4^Department of Anesthesiology and Perioperative Medicine, The Affiliated Suzhou Hospital (West District) of Nanjing Medical University, Suzhou Science and Technology Town Hospital, Suzhou 215153, China; ^5^Department of Respiratory Medicine, The Fifth Affiliated Hospital of Sun Yat-Sen University, Zhuhai, Guangdong 519000, China; ^6^Theragnostic Laboratory, Biomedical Sciences Group, KU Leuven, Leuven 3000, Belgium

## Abstract

**Purpose:**

The morbidity of esophageal adenocarcinoma (EAC) has significantly increased in Western countries. We aimed to identify trends in incidence and survival in patients with EAC in the recent 30 years and then analyzed potential risk factors, including race, sex, age, and socioeconomic status (SES).

**Methods:**

All data were collected from the Surveillance, Epidemiology, and End Results or SEER database. Kaplan–Meier analysis and the Cox proportional hazards model were conducted to compare the differences in survival between variables, including sex, race, age, and SES, as well as to evaluate the association of these factors with prognosis.

**Results:**

A total of 16,474 patients with EAC were identified from 1984 to 2013 in the United States. Overall incidence increased every 10 years from 1.8 to 3.1 to 3.9 per 100. Overall survival gradually improved (*p* < 0.0001), which was evident in male patients ((hazard ratio (HR) = 1.111; 95% confidence interval (CI) (1.07, 1.15)); however, the 5-year survival rate remained low (20.1%). The Cox proportional hazards model identified old age, black ethnicity, and medium/high poverty as risk factors for EAC (HR = 1.018; 95% CI (1.017, 1.019; HR = 1.240, 95% CI (1.151,1.336), HR = 1.000, 95% CI (1.000, 1.000); respectively).

**Conclusions:**

The incidence of EAC in the United States increased over time. Survival advantage was observed in white patients and patients in the low-poverty group. Sex was an independent prognostic factor for EAC, but this finding has to be confirmed by further research.

## 1. Introduction

The predominant histologic type of esophageal cancer globally is squamous cell carcinoma; however, in Western countries, esophageal adenocarcinoma (EAC) is the most prominent subtype [1, 2]. Approximately 17,650 new patients (13,750 males and 3,900 females) are predicted to receive the diagnosis of esophageal cancer, and 16,080 are predicted to die from this disease in the United States in 2019 [3].

In addition, EAC is a particularly fatal cancer with a poor 5-year survival rate of less than 20% [1, 4] despite advances in EAC therapies, such as endoscopic resection, radiotherapy, concurrent neoadjuvant chemoradiotherapy (NCRT) [5], and cytotoxic chemotherapy [1]. Therefore, we should not only elucidate the pathogenesis and molecular mechanisms of EAC but also analyze clinical data to strategically improve clinical management and contemporaneously enhance presymptomatic screening.

However, previous studies have analyzed the prevalence and prognosis of EAC over a short period only rather than an extended period. Some studies have only examined the outcomes of specific therapies, while others have evaluated the influence of race or sex on survival for esophageal cancer [6, 7]. Moreover, the importance of disparities in race and socioeconomic status (SES) in the healthcare system has drawn increasing attention from politicians and policy deciders in the United States. Thus, we explored the long-term trends in incidence and survival from 1984 to 2013. The aim was to evaluate the effect of race, sex, age, and SES on the prognosis of EAC by analyzing the clinical data of patients diagnosed with EAC throughout the United States, as determined from the SEER database.

## 2. Materials and Methods

### 2.1. Data Resources

A total of 16,474 patients with EAC from 1984 to 2013 were identified from the SEER database (version 8.3.5). Histologic types of EAC were determined in accordance with the International Classification of Diseases for Oncology and histologic codes (8140–8575). We excluded the patients younger than 20 years because of their extremely low incidence (2) and those diagnosed by autopsy or as stated on a death certificate.

We categorized all patients by period: 1984–1993, 1994–2003, and 2004–2013. Patient cases were also classified by sex, age, race, and SES. The median age at diagnosis was 65 years; accordingly, we subdivided age into five groups (20–44, 45–54, 55–64, 65–74, and 75+ years). The SES level was defined as in previous publications and then divided into three levels on the basis of the county poverty rate [8]. However, we integrated the medium- and high-poverty groups into medium/high poverty because of the small sample size.

### 2.2. Statistical Analyses

The two-tailed logrank test was used to access the difference in survival, using the Kaplan–Meier curves generated by the GraphPad Prism 5.0. A two-tailed *p* value < 0.05 was considered as statistically significant. The Cox proportional hazard univariate and multivariate model were used to identify survival risk factors, including sex, age, race, and SES for the entire cohort.

## 3. Results

### 3.1. Trends in Prevalence of EAC over Three Decades

**Figure 1:**
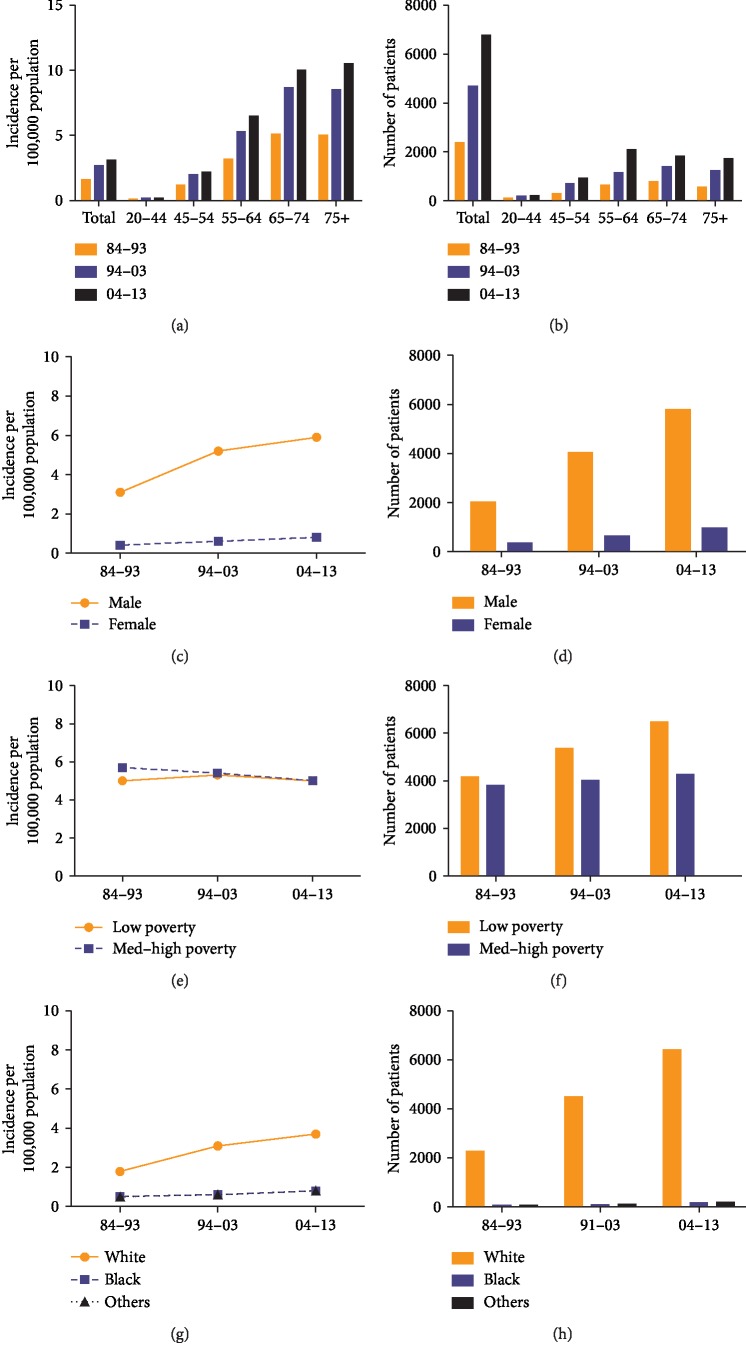
Summary incidences of patients diagnosed with EAC between 1984 and 2013 at the original nine SEER sites. Incidence (a) and number (b) of EAC cases are shown by age group (total and ages 20–44, 45–54, 55–64, 64–74, and 75+ years) and calendar period. Incidence (c), (e), (g) and number (d), (f), (h) of EAC cases are grouped by sex, race, and SES, respectively.

The overall incidence rate and the number of 16,474 patients diagnosed with EAC increased each decade over time, from 1.8 to 3.1 and 3.9 per 100,000 and from 2,715 to 5,528 and 8,231 respectively. Moreover, the incidence significantly increased with age, particularly in the age groups 65–74 and over 75 (from 6.0 in 1984–1993 to 10.4 in 1994–2003 to 12.6 in 2004–2013 and from 6.2 in 1984–1993 to 11.4 in 1994–2003 to 14.9 in 2004–2013, respectively) (Figure 1(a), [Supplementary-material supplementary-material-1] Table). Figures 1(c) and 1(d) show that compared with females, the incidence of males with EAC show a prominently higher proportion (3.5 vs. 0.5 per 100,000 in 1984–1993, 6.1 vs. 0.8 in 1994–2003, and 7.3 vs. 1.0 in 2004–2013, i.e., approximately 7.3-fold higher on the average; [Supplementary-material supplementary-material-1] Table). In addition, the overall incidence in both males and females increased over the three periods studied; the gap between them increasingly widened, which was more evidently observed in males than in females.  Figure 1(d) also shows that the number of male patients is significantly larger than that of female patients.

### 3.2. Incidence of EAC in Different Ethnicities and SES Groups

A continual increase in the incidence per 100,000 patients continually increased in all racial groups over time, with whites showing a markedly higher incidence rate than that of blacks and other ethnicities (from 2.0 to 3.7 to 4.6, respectively). However, the incidence of blacks slightly increased (from 0.5 to 0.7 to 1.0, respectively), thereby widening the incidence gap between whites and blacks (Figures 1(e) and 1(f), [Supplementary-material supplementary-material-1] Table).

A growing incidence rate for the entire period was found in all SES groups, with the highest rate recorded in the low-poverty group (from 2.1 to 3.5 to 4.1, respectively). Patients in the high-poverty group showed the lowest incidence of esophageal adenocarcinoma from 1.0 to 2.6 to 3.3 (Figures 1(g) and 1(h), [Supplementary-material supplementary-material-1]).

### 3.3. Survival for EAC Patients over Three Decades

**Figure 2:**
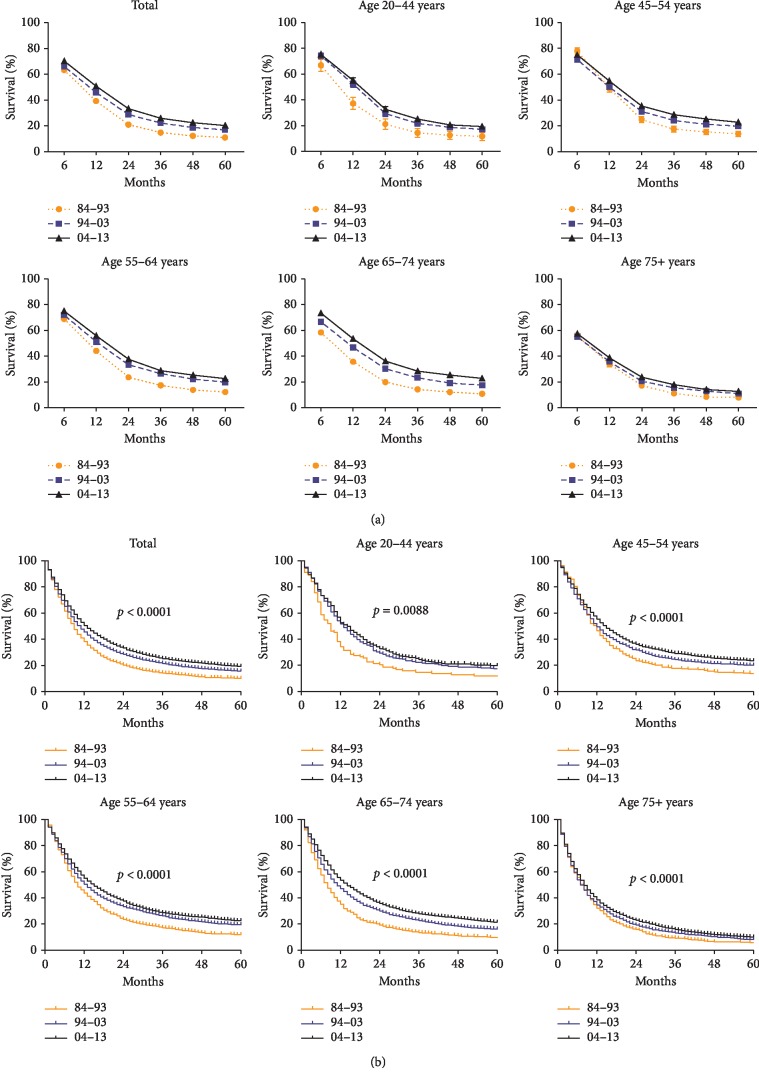
Trends in 5-year relative survival rates (a) and Kaplan–Meier survival analysis (b) for patients with EAC at eighteen SEER sites in 1984–1993 (orange), 1994–2003 (blue), and 2004–2013 (black), respectively, according to age group (total and ages 20–44, 45–54, 55–64, 64–74, and 75+ years).

As shown in Figure 2(b), prognosis is better in the recent decade than in the previous ones. This observation holds not only for the general population but also for the groups stratified by age, as determined from the Kaplan–Meier curve and the logrank test (all *p* < 0.05). The median survival for esophageal adenocarcinoma significantly improved each decade from 9 to 11 to 13 months. The 1-year relative survival rate (RSR) significantly increased from 39.2% to 45.8% to 50.8% over the three decades studied. Considering long-term survival in the study, we analyzed the 3-year survival rate, which increased from 14.7% to 22.1% to 25.8%, and the 5-year survival rates, which increased from 10.9% to 16.8% to 20.1%. This increasing tendency in overall survival over the periods studied was obtained after a 5-year follow-up (Table 1).

**Figure 3:**
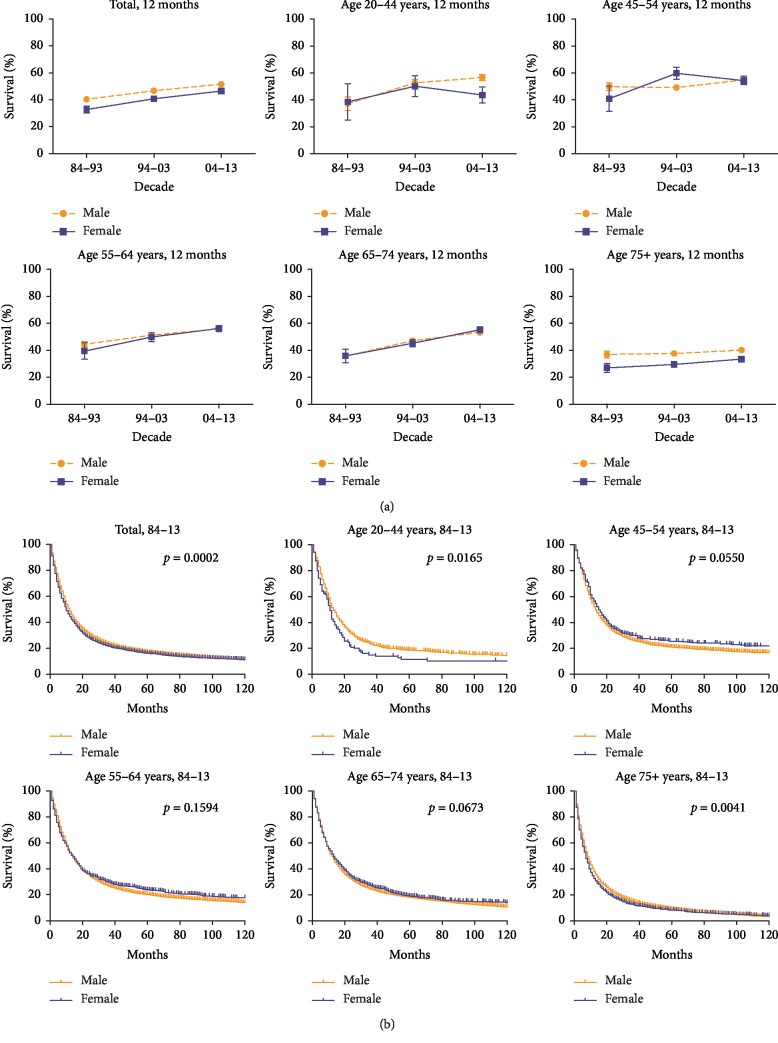
12-month relative survival rates from 1984 to 2013 (a) and Kaplan–Meier survival analysis from 1984 to 2013 (b) for male (orange) and female (blue) with EAC at eighteen SEER sites by age group (total and ages 20–44, 45–54, 55–64, 64–74, and 75+ years).

The Kaplan–Meier survival curve suggests that compared with the females, the males exhibit higher survival; however, this finding applies only for the total population and the 20–44 and over-75 age groups and not for others (Figure 3(b), *p* < 0.05). Based on the median survival over time increasing from 9 to 11 to 13 months, we chose a 12-month RSR as an indicator in the analysis of the differences in short-term survival between males and females. The outcome showed slight improvement over three decades in both males and females. However, compared with the females, the males showed a survival advantage only in total population (40.3% vs. 32.6% in 1984–1993, 46.6% vs. 40.8% in 1994–2003, and 51.5% vs. 46.5% in 2004–2013; Figure 3(a), [Supplementary-material supplementary-material-1] Table) and in the over-75 age group. Similar trends were observed in the 6- and 18-month RSRs ([Supplementary-material supplementary-material-1] Table and [Supplementary-material supplementary-material-1] Figure).

Cox models were conducted to evaluate the prognostic values of sex, race, SES, and age for EAC over the study periods. Both univariate and multivariate analyses revealed that all variables, except for sex, could serve as potential predictors of prognosis. Univariate analysis determined that sex was an independent predictor (HR = 1.111; 95% CI (1.07, 1.15)), whereas multivariate analysis indicated that sex was not associated with prognosis (HR = 1.028; 95% CI (0.99, 1.07); Table 2).

### 3.4. EAC Survival in Race and SES

**Figure 4:**
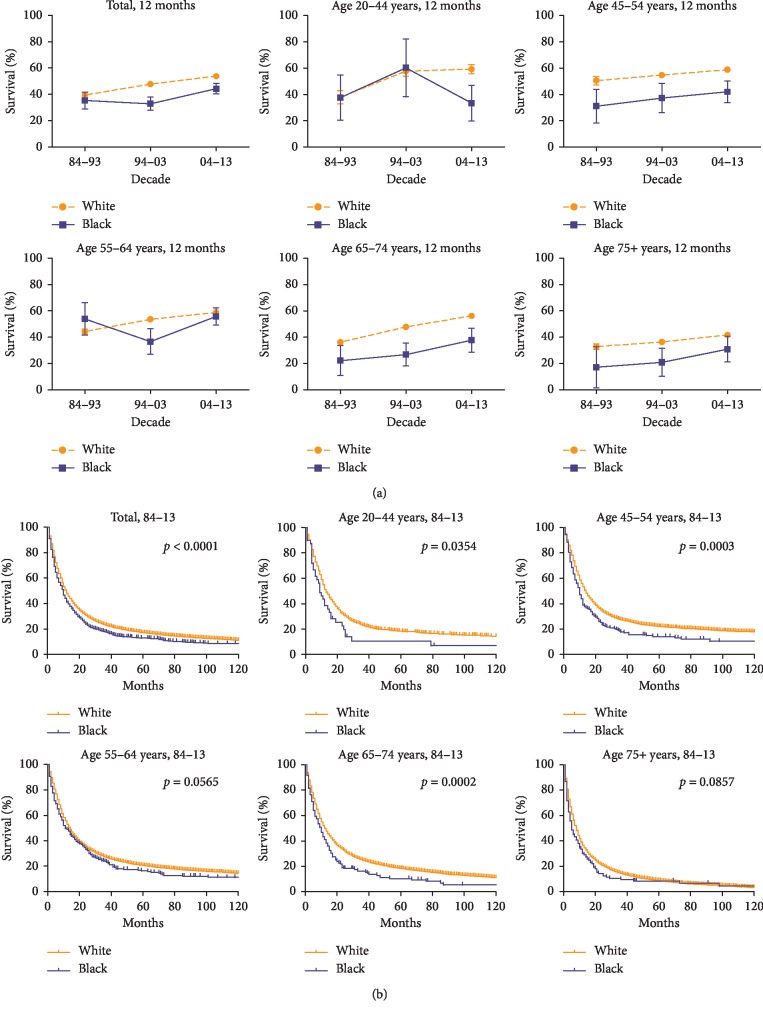
12-month relative survival rates from 1984 to 2013 (a) and Kaplan–Meier survival analysis from 1984 to 2013 (b) for white (orange) and black (blue) with EAC at eighteen SEER sites by age group (total and ages 20–44, 45–54, 55–64, 64–74, and 75+ years).

Black patients with EAC had worse survival than whites in all populations over three 10-year periods, as verified and confirmed by Kaplan–Meier survival curves and logrank test with *p* < 0.0001 (Figure 4(b)). This disadvantage for blacks was also found in the 20–44 (*p*=0.035), 45–54 (*p* < 0.001), and 65–74 (*p* < 0.001) age groups. Analysis of the 12-month RSR in 1984–1993 showed that black patients had slightly lower RSRs than those of white patients (39.3% vs. 36.2%; [Supplementary-material supplementary-material-1] Table). This survival advantage for white patients became evident in the second period when the survival rate of whites increased, whereas that of blacks decreased (46.1% vs. 35.6%). However, the survival gap decreased again because the survival advantage significantly increased for black patients in the third period (51.0% for whites vs. 45.6% for blacks; Figure 4(a) and [Supplementary-material supplementary-material-1] Table). Similar but less pronounced changes were detected in 6- and 18-month RSRs for white and black patients ([Supplementary-material supplementary-material-1] Figure and [Supplementary-material supplementary-material-1] Table).

**Figure 5:**
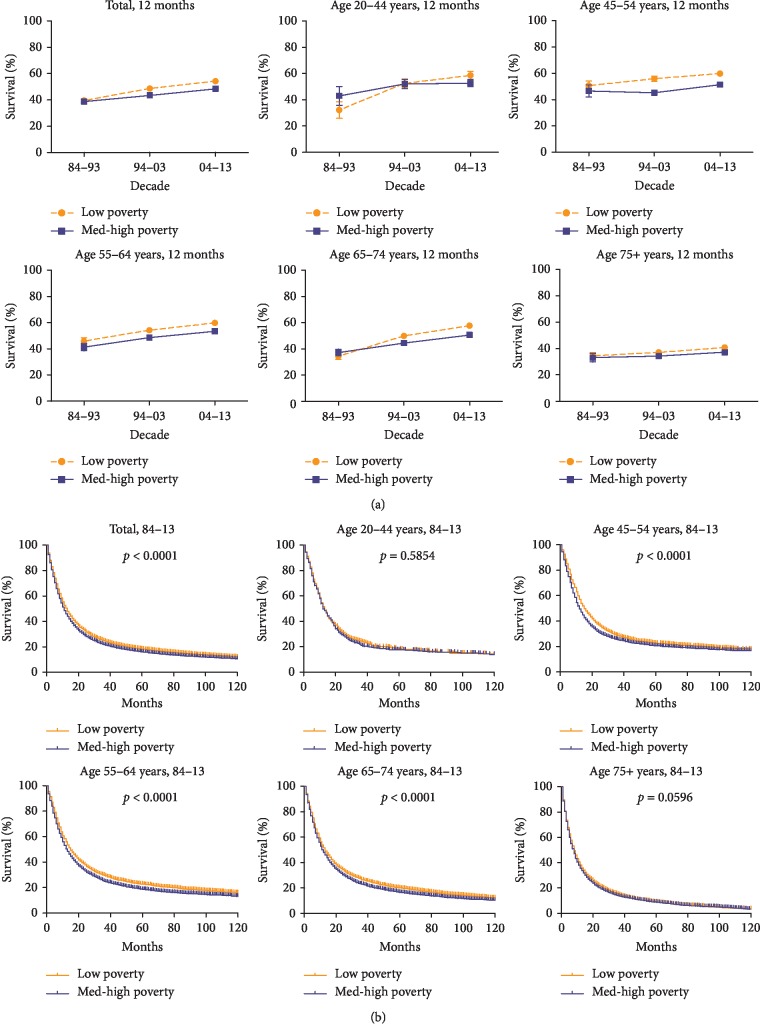
12-month relative survival rates from 1984 to 2013 (a) and Kaplan–Meier survival analysis from 1984 to 2013 (b) for low-poverty (orange) and med-high-poverty (blue) with EAC at eighteen SEER sites by age group (total and ages 20–44, 45–54, 55–64, 64–74, and 75+ years).

When we divided SES into three levels—low, medium, and high poverty—and then stratified the sample by age groups, the sample sizes of the middle and high-poverty groups in each age group were found to be too small, increasing the standard error of the mean. Consequently, we integrated the medium-poverty and high-poverty groups into one: the medium/high-poverty group.  Figure 5(b) shows that the patients in the low-poverty group survive better, compared with the patients in the medium/high-poverty group. A slight improvement in the 12-month RSR was observed in both SES groups over the periods studied (from 39.5% to 48.7% to 54.3% for the low-poverty group; from 38.8% to 43.4% to 48.4% for the medium/high-poverty group; Figure 5(a), [Supplementary-material supplementary-material-1] Table). As shown in Figure 5(a) and the aforementioned statistics, the gap in survival rate between the low-poverty group and the medium/high-poverty group continued to widen over time, increasing the differences between them from 0.7% to 4.7% to 5.9%. Similar increases in survival and disparities in SES were also found in the 6- and 18-month RSRs ([Supplementary-material supplementary-material-1] Figure and [Supplementary-material supplementary-material-1] Table). The proportions of disparity in the SES groups varied between whites and blacks: patients in the low-poverty group comprised 63.8% of whites but only 25.6% of blacks and more patients in the medium/high-poverty group were distributed among blacks than whites (74.4% vs. 36.2%, [Supplementary-material supplementary-material-1] Figure, [Supplementary-material supplementary-material-1] Table). Moreover, both univariate and multivariate Cox analyses suggested that SES affected the survival of patients with EAC (HR = 1.000, 95% CI (1.000–1.000), Table 2).

## 4. Discussion

In the general population, the prevalence of EAC continued to accelerate every decade from 1984 to 2013 in the United States. As for survival advantage, the median survival improved every decade from 9 to 11 to 13 months and the 5-year survival rate ultimately exceeded 20% by a marginal percentage (20.1%).

The overall incidence of EAC significantly increased each decade from 1.8 to 3.1 to 3.9 per 100,000 and was predominant in patients older than 65 years. Meanwhile, compared with females, males were more prone to develop EAC, that is, almost 7.6-fold higher in risk on average over the three decades studied. The incidence gap between them widened over time, with a more evident increase in males than in females. Compared with blacks and other ethnicities, whites were more prone to develop EAC, and the morbidity gap between them widened decade by decade in the 30-year period studied. A continuously growing incidence rate was found in all SES groups during the entire study periods. The highest proportion of morbidity was found in the low-poverty group (from 1.8 to 3.0 to 3.3 per 100,000, respectively).

However, the mechanism underlying the different disparities between gender and race on the prevalence of EAC remains less understood. One hypothesis was that sex steroid hormones exerted an effect on gender difference in Barrett's esophagus (BE) and EAC prevalence [9]. Genetic variants [10, 11] and other prominent etiological factors encompassing BE (neoplastic precursor lesion of EAC), gastroesophageal reflux disease (GERD) [12, 13], and *Helicobacter pylori* infection (inverse relation to BE [14] and EAC [15, 16]) played a dominant role in EAC development. Interestingly, it was found that it is abdominal obesity or waist-to-hip ratio (WHR) which are predominantly distributed in white men [17], rather than body mass index (BMI) resulting in hormonal, adipokine, and cytokine alterations and then causing BE and EAC [18]. A case-control study reported that non-Hispanic whites (NHWs) were more likely to have a high WHR and use proton-pump inhibitors (PPIs; a decreased risk of progression to EAC [19]) and hiatal hernia, but less prone to have *Helicobacter pylori* infection than African Americans (AAs) [20], which might to some extent contributed to the racial disparity in prevalence of EAC. Gender- and race-specific susceptibilities to EAC could be interpreted only when all aforementioned elements were to be comprehensively considered.

Overall survival and 1-, 3-, and 5-year survival for patients with EAC significantly improved in the general population and the over-30 age group. Median survival also improved from 9 to 11 to 13 months. These increments could be attributed to advances in clinical treatment and management of patients with EAC in the past decades, such as the implementation of concurrent NCRT [5, 21] and cytotoxic chemotherapy. However, the 5‐year survival rate improved only slightly from 10.9% to 16.8% to 20.1% (slightly more than 20%), indicating an urgent need for further research and development of novel treatment to prevent deterioration and metastasis.

With respect to the survival difference in sex, the 6-, 12-, and 18-month RSRs slightly improved in both males and females, with the males exhibiting survival advantage over women in the total population and the over-75 age group over the three periods studied. However, this survival advantage for males was eliminated when stratification by age was conducted. Kaplan–Meier survival analysis indicated that no difference in survival existed between male and female patients with EAC (*p*=0.1367 in 1984–1993; *p*=0.0152 in 1994–2003; *p*=0.0174 in 2004–2013; Figure 3). The Cox proportional hazards regression models also verified that the association between sex and prognosis was not relevant for EAC (HR = 1.028; 95% CI (0.99, 1.07)). A previous study confirmed that sex was not a crucial prognostic factor for EAC [7]. However, further research is needed to confirm the survival advantage of males over females.

During the period from 1984 to 1993, the 12-month RSR was higher for white patients than for black patients (39.3% vs. 36.2%; Figure 4(a) and [Supplementary-material supplementary-material-1]). This survival superiority for the whites became remarkable with the decrease in survival rate in blacks during the second period (46.1% vs. 35.6%). However, the survival gap was reduced again when the survival rate in blacks increased during the third period (51.0% for whites vs. 45.6% for blacks). The blacks were diagnosed predominantly with squamous carcinoma at an advanced stage with more comorbidities and were less likely to receive surgical resection which might contribute to poorer survival than that of whites [22, 23]. These reasons could also partly explain the racial inequalities in the survival of EAC. The slight improvement in survival for black and the reduced gap during the third period could be attributable to the better standard of living and greater access to prompt treatment than those in the previous two periods.

Consistently, the medium/high-poverty group showed worse survival than that of the low-poverty group, and the gap in 12-month RSR between them continued to widen over time. Notably, white patients were mostly classified in the low-poverty group (63.8%), whereas black patients were mostly classified in the medium/high-poverty group (74.4%). This difference in SES between ethnicities, consistent with the poorer survival for blacks than for whites, might contribute to the widening survival gap between the low-poverty group and the medium/high-poverty group.

To present more realistically the morbidity and mortality of EAC in the United States, we excluded the patients younger than 20 years, considering that the sample size of this age group was extremely small (2) that it increased the standard error of the mean. We disregarded the effect of stage or therapy on overall survival as such was not the main objective of this study. In addition, the clinical treatment and TNM staging systems [24, 25] had changed over time. No data on changes in individual economic status and insurance status were available in the SEER registry; thus, caution should be exercised when interpreting and applying these results and drawing conclusions in healthcare policy design and other areas. Moreover, this study might be affected by bias, under-registration, and misclassification in the SEER database.

## 5. Conclusions

The incidence of EAC increased over the past 30 years and would predictably continue in the future. The overall survival significantly improved each decade, but the 5-year survival rate remained low (20.1%). Here, we demonstrated the disparities in both incidence and survival: with a higher incidence in men and whites and poorer survival in blacks and patients lived in medium/high-poverty regions. And great attention is necessitated to increase public awareness by education and promote early diagnosis, which ultimately helps improve survival. Specific susceptibilities to EAC in male and white patients might at least partly result from the similarity in sex and race susceptibilities to GERD and BE. Therefore, effective prevention of GERD and BE might contribute to the decrease in prevalence of EAC. Knowing the incidence and survival tendencies of EAC and their disparities between race, sex, age, and SES, the government could also introduce new healthcare measures to reduce morbidity and improve prognosis. Furthermore, this study can potentially guide further studies on the molecular mechanisms of sex and race disparities in EAC.

## Figures and Tables

**Table 1:** Relative survival rates of EAC during 1984–1993, 1994–2003, and 2004–2013 at eighteen SEER sites.

Age group	Decade		
	1984–1993	1994–2003	2004–2013
6-Mo RSR			
All	63.2 ± 1 (2584)	66.0 ± 0.5 (9196)^*∗*^	70.3 ± 0.4 (18111)^*∗∗∗*^
20–44	66.8 ± 4.6 (105)	74.1 ± 2.2 (409)	75.3 ± 1.8 (622)
45–54	78.0 ± 2.4 (311)	71.4 ± 1.2 (1398)^*∗*^	75.2 ± 0.9 (2550)^*∗*^
55–64	68.8 ± 1.8 (699)	71.9 ± 0.9 (2316)^*∗∗∗*^	75.0 ± 0.6 (5469)^*∗*^
65–74	58.3 ± 1.7 (852)	66.6 ± 0.9 (2692)^*∗∗∗*^	73.4 ± 0.7 (4946)^*∗∗∗*^
75+	55.5 ± 2.1 (617)	55.0 ± 1.1 (2381)	57.5 ± 0.8 (4524)

12-Mo RSR			
All	39.2 ± 1.0	45.8 ± 0.5^*∗∗∗*^	50.8 ± 0.4^*∗∗∗*^
20–44	37.2 ± 4.7	52.3 ± 2.5^*∗*^	55.0 ± 2.1
45–54	49.0 ± 2.9	50.0 ± 1.3	54.8 ± 1.0^*∗*^
55–64	44.0 ± 1.9	51.1 ± 1.1^*∗*^	55.9 ± 0.7^*∗∗*^
65–74	35.6 ± 1.7	46.8 ± 1.0^*∗∗∗*^	53.5 ± 0.8^*∗∗∗*^
75+	33.9 ± 2.0	35.6 ± 1.0	38.6 ± 0.8^*∗*^

24-Mo RSR			
All	20.9 ± 0.8	28.7 ± 0.5^*∗∗∗*^	33.3 ± 0.4^*∗∗∗*^
20–44	21.1 ± 4	29.3 ± 2.3	32.7 ± 2
45–54	24.8 ± 2.5	31.1 ± 1.2^*∗*^	35.4 ± 1^*∗∗*^
55–64	23.5 ± 1.6	33.3 ± 1^*∗∗∗*^	37.6 ± 0.7^*∗∗*^
65–74	19.8 ± 1.4	30.2 ± 0.9^*∗∗∗*^	36.1 ± 0.8^*∗∗∗*^
75+	17.1 ± 1.7	20.5 ± 0.9	23.7 ± 0.7^*∗*^

36-Mo RSR			
All	14.7 ± 0.7	22.1 ± 0.5^*∗∗∗*^	25.8 ± 0.4^*∗∗∗*^
20–44	14.4 ± 3.4	21.7 ± 2.1	25.1 ± 1.9
45–54	17.4 ± 2.2	24.1 ± 1.2^*∗*^	28.6 ± 1^*∗*^
55–64	17.3 ± 1.5	26.4 ± 0.9^*∗∗∗*^	28.8 ± 0.7
65–74	14.2 ± 1.3	23.1 ± 0.9^*∗∗∗*^	28.3 ± 0.8^*∗∗∗*^
75+	11.0 ± 1.5	15.4 ± 0.9^*∗*^	17.9 ± 0.7^*∗*^

48-Mo RSR			
All	12.1 ± 0.7	18.6 ± 0.4^*∗∗∗*^	22.4 ± 0.4^*∗∗∗*^
20–44	12.5 ± 3.2	18.7 ± 2	20.5 ± 1.8
45–54	15.2 ± 2.1	21.0 ± 1.1^*∗*^	25.2 ± 1^*∗*^
55–64	13.7 ± 1.4	22.1 ± 0.9^*∗∗∗*^	25.2 ± 0.7^*∗*^
65–74	12.0 ± 1.2	19.1 ± 0.8^*∗∗∗*^	25.3 ± 0.8^*∗∗∗*^
75+	8.3 ± 1.3	12.8 ± 0.8^*∗*^	14.1 ± 0.7

60-Mo RSR			
All	10.9 ± 0.7	16.8 ± 0.4^*∗∗∗*^	20.1 ± 0.4^*∗∗∗*^
20–44	11.6 ± 3.1	17.0 ± 1.9	19.4 ± 1.8
45–54	13.7 ± 2	19.7 ± 1.1^*∗*^	22.7 ± 1
55–64	12.1 ± 1.3	19.7 ± 0.9^*∗∗∗*^	22.6 ± 0.7^*∗*^
65–74	10.7 ± 1.2	17.4 ± 0.8^*∗∗∗*^	22.7 ± 0.8^*∗∗∗*^
75+	7.9 ± 1.3	10.9 ± 0.8	12.6 ± 0.7

Data are mean ± standard error of the mean, with number of patients in parentheses. Mo, month; RSR, relative survival rate; SEM, standard error of the mean. ^*∗*^*p* < 0.05, ^*∗∗*^*p* < 0.001, and ^*∗∗∗*^*p* < 0.0001 for comparisons with the preceding decade.

**Table 2:** Cox regression analysis of survival in patients with EAC from 1984 to 2013.

	Variable	Relative risk (95% CI)	*p* value
All 1984–2013			
	Univariable		
	Sex	1.111 (1.072–1.151)	<0.001
	Age	1.018 (1.017–1.019)	<0.001
	Race	1.197 (1.112–1.228)	<0.001
	SES	1.000 (1.000–1.000)	<0.001
	Multivariate		
	Sex	1.028 (0.992–1.065)	0.132
	Age	1.018 (1.017–1.019)	<0.001
	Race	1.240 (1.151–1.336)	<0.001
	SES	1.000 (1.000–1.000)	<0.001

All 1984–1993			
	Univariable		
	Sex	1.159 (1.038–1.295)	0.009
	Age	1.016 (1.012–1.019)	<0.001
	Race	1.135 (0.890–1.447)	0.307
	SES	1.000 (0.9999–1.000)	0.925
	Multivariate		
	Sex	1.052 (0.940–1.178)	0.376
	Age	1.015 (1.012–1.019)	<0.001

All 1994–2003			
	Univariable		
	Sex	1.118 (1.053–1.188)	<0.001
	Age	1.018 (1.017–1.021)	<0.001
	Race	1.308 (1.153–1.484)	<0.001
	SES	1.000 (1.000–1.000)	<0.001
	Multivariate		
	Sex	1.017 (0.956–1.081)	0.600
	Age	1.019 (1.017–1.021)	<0.001
	Race	1.313 (1.156–1.492)	<0.001
	SES	1.000 (1.000–1.000)	<0.001

All 2004–2013			
	Univariable		
	Sex	1.096 (1.045–1.149)	<0.001
	Age	1.017 (1.016–1.019)	<0.001
	Race	1.151 (1.043–1.269)	0.005
	SES	1.000 (1.000–1.000)	<0.001
	Multivariate		
	Sex	1.028 (0.980–1.079)	0.251
	Age	1.018 (1.016–1.019)	<0.001
	Race	1.192 (1.080–1.315)	0.001
	SES	1.000 (1.000–1.000)	<0.001

95% CI, 95% confidence interval; SES, socioeconomic status.

## Data Availability

The data used in this study are available from the corresponding author on reasonable request.

## Abbreviations

EAC: Esophageal adenocarcinoma
BE: BARRETT'S esophagus
SES: Socioeconomic status
RSR: Relative survival rate
HR: HAZARD ratio
CI: Confidence interval
SEER: The Surveillance, Epidemiology, and End Results.

## Supplementary Materials

Supplementary Materials 6-month (a) and 18-month (b) relative survival rates for patients with EAC at nine SEER sites from 1984 to 2013 according to sex and age group (total and ages 20–44, 45–54, 55–64, 64–74, and 75+ years) are shown in Figure S1, as described in part of “trends in prevalence of EAC over three decades” . Figure S2: 6-month (a) and 18-month (b) relative survival rates for patients with EAC at nine SEER sites from 1984 to 2013 according to race and age group (total and ages 20–44, 45–54, 55–64, 64–74, and 75+ years), as supplementary analysis of 12-month (b) relative survival rates. Figure S3: 6-month (a) and 18-month (b) relative survival rates for patients with EAC at nine SEER sites from 1984 to 2013 according to SES and age group (total and ages 20–44, 45–54, 55–64, 64–74, and 75+ years), as described in part of “Survival for EAC patients over three decades” of manuscript. Figure S4: distribution of SES by race for patients with EAC at nine SEER sites during 1984–2013, 1984–1993, 1994–2003, and 2004–2013, respectively. Percentage (a) and number (b) of patients with EAC in low-poverty and med-high-poverty groups, described in part of “EAC survival in race and SES.” Supplementary tables show the incidence and relative survival rates of EAC patients according to sex, age, and three decades and the summary data for race distribution by SES.
