# Supplementary material for: Incidence and Survival Changes in Patients with Esophageal Adenocarcinoma during 1984–2013
Source: Biomed Res Int. 2019 Dec 12;2019:7431850. doi: 10.1155/2019/7431850 (PMC6930790; doi:10.1155/2019/7431850)
Supplement: Supplementary Materials — 6-month (a) and 18-month (b) relative survival rates for patients with EAC at nine SEER sites from 1984 to 2013 according to sex and age group (total and ages 20–44, 45–54, 55–64, 64–74, and 75+ years) are shown in Figure S1, as described in part of “trends in prevalence of EAC over three decades” . Figure S2: 6-month (a) and 18-month (b) relative survival rates for patients with EAC at nine SEER sites from 1984 to 2013 according to race and age group (total and ages 20–44, 45–54, 55–64, 64–74, and 75+ years), as supplementary analysis of 12-month (b) relative survival rates. Figure S3: 6-month (a) and 18-month (b) relative survival rates for patients with EAC at nine SEER sites from 1984 to 2013 according to SES and age group (total and ages 20–44, 45–54, 55–64, 64–74, and 75+ years), as described in part of “Survival for EAC patients over three decades” of manuscript. Figure S4: distribution of SES by race for patients with EAC at nine SEER sites during 1984–2013, 1984–1993, 1994–2003, and 2004–2013, respectively. Percentage (a) and number (b) of patients with EAC in low-poverty and med-high-poverty groups, described in part of “EAC survival in race and SES.” Supplementary tables show the incidence and relative survival rates of EAC patients according to sex, age, and three decades and the summary data for race distribution by SES. [file 7431850.f1.pdf]

Incidence and survival changes in patients with esophageal adenocarcinoma during 1984-2013

Haiyu Zhang [1,*], Xiaofeng Pei [2,*], Xiangqiong Mo [3], Junlan Qiu [4], Xiaobin Zheng[5], Shuncong Wang [6], Huanhuan Sun [1,#], Haiqing Ma [1,#]

[1] Department of Department of Oncology, The Fifth Affiliated Hospital of Sun Yat-Sen University, Zhuhai, Guangdong 519000, China;

[2] Department of Thoracic Oncology, The Fifth Affiliated Hospital of Sun Yat-Sen University, Zhuhai, Guangdong 519000, China;

[3] Department of Gastrointestinal Surgery, MD, The Fifth Affiliated Hospital of Sun Yat-Sen University, Zhuhai, Guangdong 519000, China;

[4] Department of Anesthesiology and Perioperative Medicine, The Affiliated Suzhou Hospital (West District) of Nanjing Medical University; Suzhou Science and Technology Town Hospital, Suzhou 215153, China;

[5] Department of Respiratory Medicine, The Fifth Affiliated Hospital of Sun Yat-Sen University, Zhuhai, Guangdong 519000, China;

[6] Theragnostic Laboratory, Biomedical Sciences Group, KU Leuven, Leuven 3000, Belgium.

***These authors have contributed equally to this work.**

#Correspondence to: Huanhuan Sun email: sunhuanh3@mail.sysu.edu.cn

Hai-Qing Ma, email: mahaiqing@mail.sysu.edu.cn

Telephone Number: +86-756-2528888

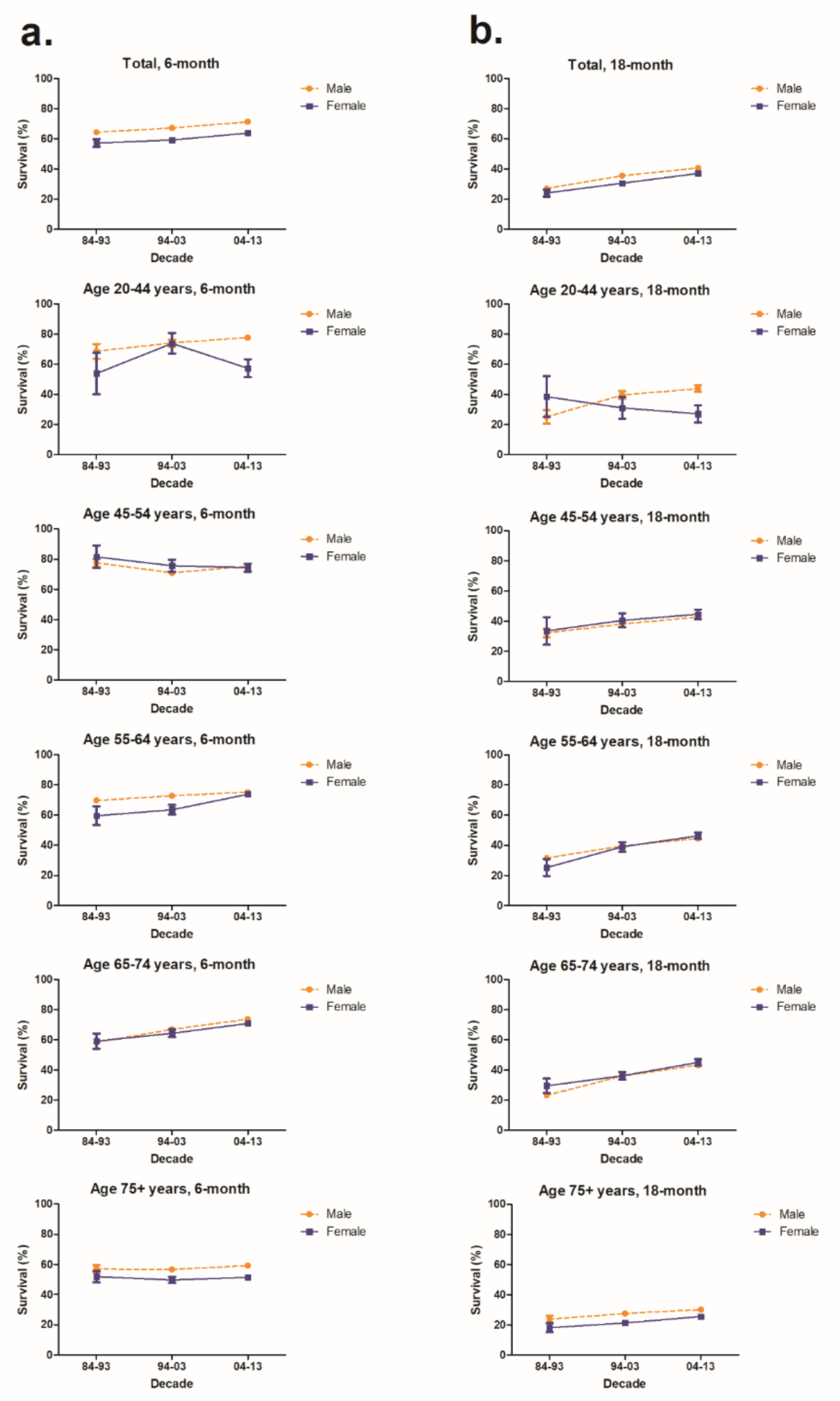

Figure S1. 6-month (a) and 18-month (b) relative survival rates for patients with EAC at nine SEER sites from 1984 to 2013 according to sex and age group (total and ages 20-44, 45-54, 55–64, 64-74 and 75+ years).

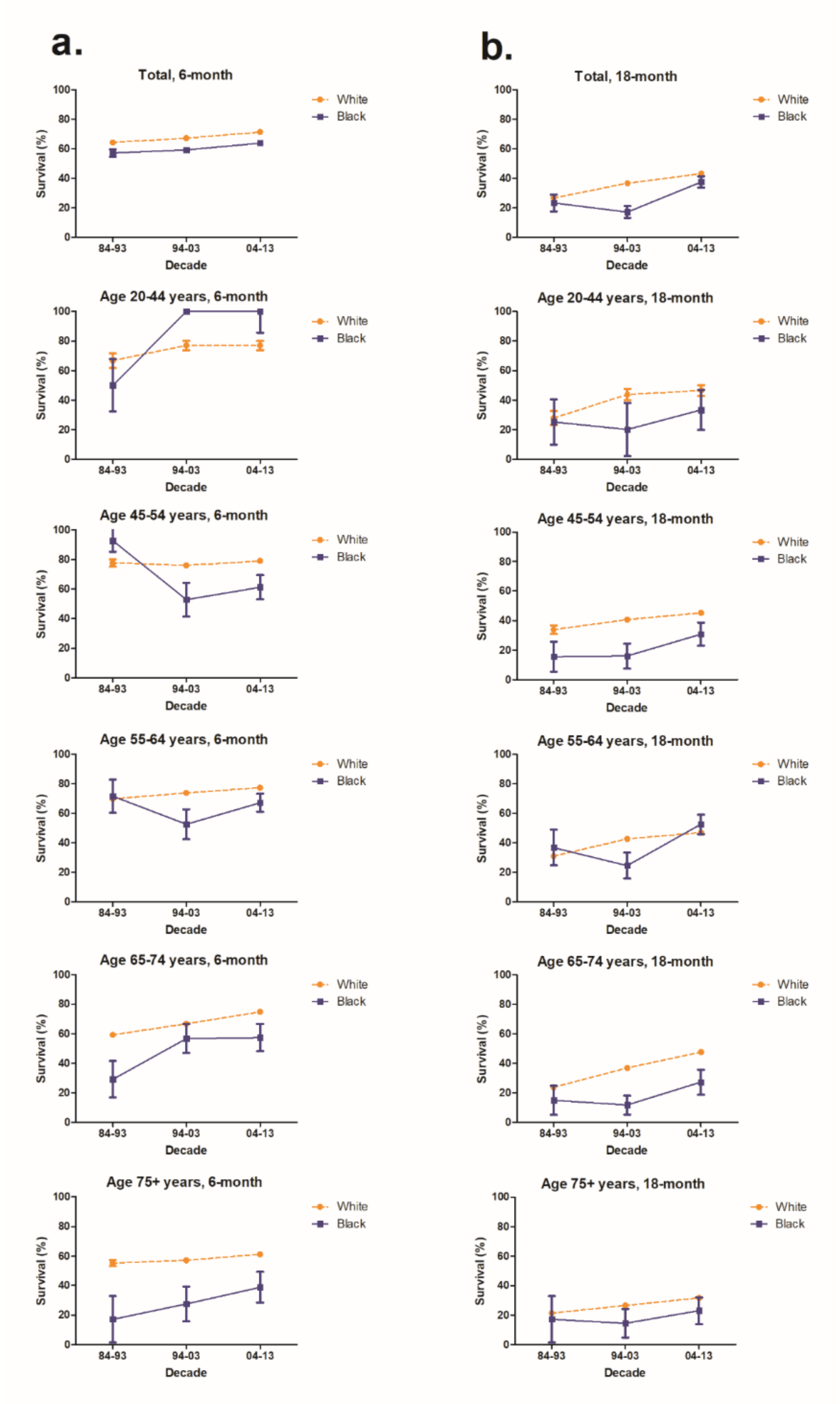

Figure S2. 6-month (a) and 18-month (b) relative survival rates for patients with EAC at nine SEER sites from 1984 to 2013 according to race and age group (total and ages 20-44, 45-54, 55–64, 64-74 and 75+ years).

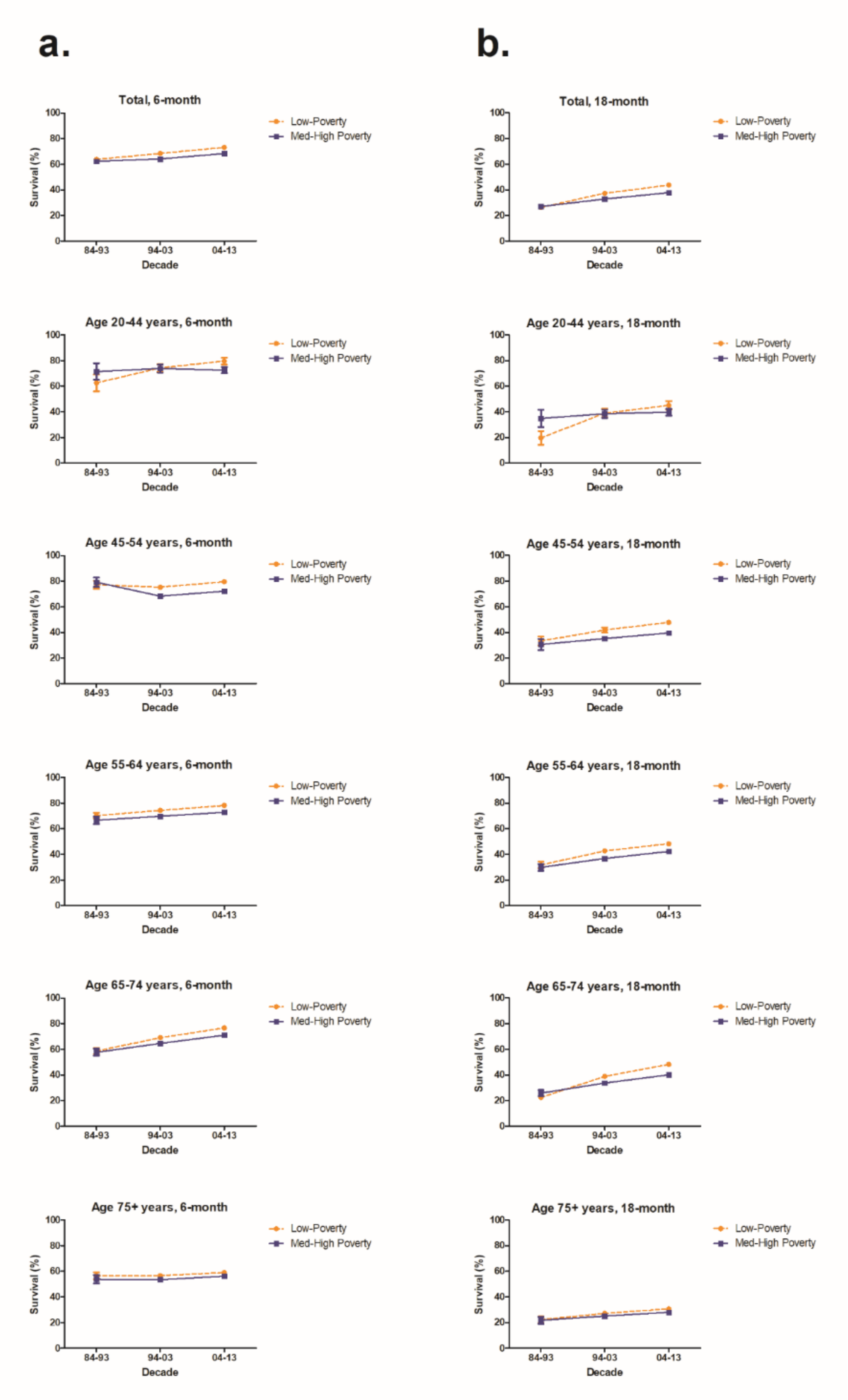

Figure S3. 6-month (a) and 18-month (b) relative survival rates for patients with EAC at nine SEER sites from 1984 to 2013 according to SES and age group (total and ages 20-44, 45-54, 55–64, 64-74 and 75+ years).

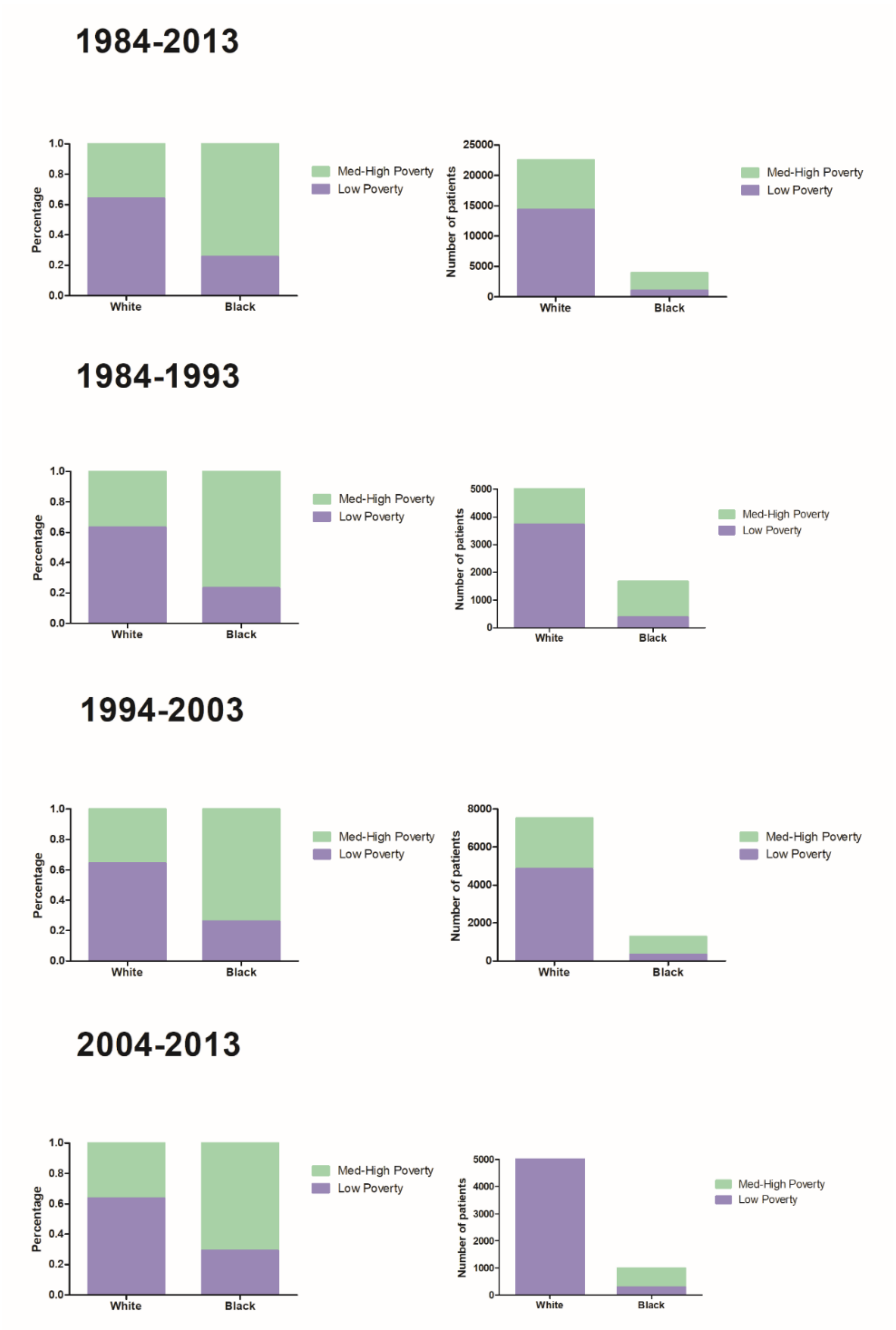

Figure S4. Distribution of SES by race for patients with EAC at nine SEER sites during 1984-2013, 1984-1993, 1994-2003 and 2004-2013, respectively. Percentage (a) and number (b) of patients with EAC in Low-poverty, Med-High-Poverty groups.

Table S1. **The incidence of EAC according to age and decade within sex, SES, and race groups.**

Variable		Age Groups	Decade		
			1984-1993	1994-2003	2004-2013
Total					
		All	1.8 (2715)	3.1 (5528)	3.9 (8231)
		20-44	0.1 (104)	0.2 (205)	0.2 (219)
		45-54	1.3 (298)	2.1 (719)	2.3 (964)
		55-64	3.4 (683)	5.8 (1255)	7.1 (2293)
		65-74	6.0 (937)	10.4 (1683)	12.6 (2296)
		75+	6.2 (693)	11.4 (1666)	14.9 (2459)
Sex	Male				
		All	3.5 (2308)	6.1 (4717)	7.3 (7036)
		20-44	0.2 (90)	0.4 (182)	0.4 (196)
		45-54	2.4 (272)	3.9 (662)	4.0 (829)
		55-64	6.5 (618)	10.7 (1118)	13.1 (2039)
		65-74	12.0 (827)	20.4(1479)	23.7 (1997)
		75+	12.4 (501)	23.5 (1276)	30.6 (1975)
	Female				
		All	0.5 (407)	0.8 (811)	1.0 (1195)
		20-44	0.0 (14)	0.0(23)	0.0 (23)
		45-54	0.2 (26)	0.3 (57)	0.6 (135)
		55-64	0.6 (65)	1.2 (137)	1.5 (254)
		65-74	1.3 (110)	2.3 (204)	3.1 (299)
		75+	2.6 (192)	4.2 (390)	4.6 (484)
Race	White				
		All	2.0 (2601)	3.7 (5307)	4.6 (7802)
		20-44	0.1 (93)	0.3 (192)	0.3 (196)
		45-54	1.4 (281)	2.5 (685)	2.8 (888)
		55-64	3.9 (652)	6.9 (1202)	8.5 (2158)
		65-74	6.7 (904)	12.2 (1616)	15.1 (2200)
		75+	6.8 (571)	12.9 (1612)	17.4 (2360)
	Black				
		All	0.5 (65)	0.7 (107)	1.0 (202)
		20-44	0.1 (8)	0.0 (5)	0.1 (12)
		45-54	0.6 (13)	0.6 (21)	0.8 (38)
		55-64	1.2 (19)	1.4 (27)	2.1 (69)
		65-74	1.5 (18)	2.7 (34)	2.7 (44)
		75+	1.0 (7)	2.1 (20)	3.4 (39)
	Other				
		All	0.5 (49)	0.7 (114)	0.9 (227)
		20-44	0.0 (3)	0.1 (8)	0.1 (11)
		45-	0.2 (4)	0.4 (13)	0.8 (38)

		54			
		55-64	0.8 (12)	1.2 (26)	1.8 (66)
		65-74	1.4 (15)	2.1 (33)	2.5 (52)
		75+	2.5 (15)	3.1 (34)	3.5 (60)
SES	Low Poverty				
		All	2.1 (1713)	3.5 (3539)	4.1 (5236)
		20-44	0.1 (58)	0.3 (146)	0.3 (141)
		45-54	1.5 (197)	2.2 (443)	2.4 (607)
		55-64	3.9 (431)	6.6 (826)	7.4 (1427)
		65-74	6.8 (585)	11.4 (1039)	13.5 (1461)
		75+	7.1 (442)	12.8 (1085)	16.1 (1600)
	Medium Poverty				
		All	1.5 (968)	2.7 (1868)	3.5 (2802)
		20-44	0.1 (45)	0.1 (54)	0.2 (76)
		45-54	1.0 (96)	1.9 (262)	2.2 (340)
		55-64	2.9 (245)	4.8 (406)	6.7 (812)
		65-74	5.2 (343)	9.1 (597)	11.3 (773)
		75+	5.0 (239)	9.6 (549)	13.3 (801)
	High Poverty				
		All	1.0 (34)	2.6 (121)	3.3 (193)
		20-44	0.1 (1)	0.2 (5)	0.1 (2)
		45-54	0.9 (5)	1.7 (14)	1.6 (17)
		55-64	1.4 (7)	3.8 (23)	6.3 (54)
		65-74	2.5 (9)	10.2 (47)	11.0 (62)
		75+	4.8 (12)	9.4 (32)	13.3 (58)

Data are incidence per 100,000 people by year of diagnosis, with the number of patients in parentheses

Table S2. **12-month relative survival rates of EAC patients according to sex, age and three decades.**

Decade	Age Group	Sex	
		Male	Female
84-93	12-Mo RSR		
	All	40.3±1.1 (2202)	32.6±2.5(382) ***
	20-44	37.1±5.0 (92)	38.5±13.5(13)
	45-54	49.8±3.0 (284)	40.9±9.5(27)
	55-64	44.4±2 (635)	39.4±6.1(64)
	65-74	35.6±1.8 (758)	35.8±5(94)
	75+	36.9±2.5 (433)	26.9±3.4(184) *
94-03	12-Mo RSR		
	All	46.6±0.6 (7855)	40.8±1.4 (1341) ***
	20-44	52.5±2.6 (367)	50.1±7.7(42)
	45-54	49.1±1.4 (1279)	59.8±4.5(119) *
	55-64	51.2±1.1 (2074)	49.8±3.3(242)
	65-74	47.1±1.1 (2340)	45.2±2.7(352)
	75+	37.5±1.2 (1795)	29.5±2(586) **
04-13	12-Mo RSR		
	All	51.5±0.4 (15619)	46.5±1.1 (2492) ***
	20-44	56.6±2.2 (549)	43.6±6(73) *
	45-54	54.8±1.1 (2277)	54.4±3.1(273)
	55-64	55.9±0.7 (4908)	56.2±2.2(561)
	65-74	53.3±0.8(4345)	55.2±2.1(601)
	75+	40.1±0.9(3540)	33.4±1.6(984) ***

Data are means ± standard error of the mean, with number of patients in parentheses
Abbreviations: Mo, month; RSR, relative survival rate SEM, standard error of the mean.
*$p < 0.05$, ** $p < 0.001$, and *** $p < 0.0001$ for comparisons with the Male group.

Table S3. 6-month and 18-month relative survival rates of EAC patients according to sex, age and three decades.

Decade	Age Group	Sex	
		Male	Female
84-93	6-Mo RSR		
	All	64.3 ± 1.0 (2202)	57.2 ± 2.6(382) *
	20-44	68.6 ± 4.9 (92)	53.9 ± 13.8 (13)
	45-54	77.6 ± 2.5 (284)	81.6 ± 7.5 (27)
	55-64	69.7 ± 1.8 (635)	59.6 ± 6.2 (64)
	65-74	58.3 ± 1.8 (758)	59.1 ± 5.1 (94)
	75+	57.0 ± 2.5 (433)	52.0 ± 3.8 (184)
	18-Mo		
	All	26.9 ± 1.0 (2017)	24.1 ± 2.4(351)
	20-44	26.5 ± 4.7 (87)	38.5 ± 13.5 (13)
	45-54	32.6 ± 2.9 (263)	36.2 ± 9.7 (25)
	55-64	31.9 ± 2.0 (577)	23.1 ± 5.6 (57)
	65-74	23.1 ± 1.7 (698)	28.1 ± 4.9 (88)
	75+	22.2 ± 2.3 (392)	19.3 ± 3.3 (168)
94-03	6-Mo RSR		
	All	67.2 ± 0.5 (7855)	59.2 ± 1.4 (1341) ***
	20-44	74.1 ± 2.3 (367)	73.9 ± 6.8 (42)
	45-54	71.0 ± 1.3 (1279)	75.7 ± 3.9 (119)
	55-64	72.8 ± 1.0 (2074)	63.5 ± 3.1 (242) *
	65-74	66.9 ± 1.0 (2340)	64.4 ± 2.6 (352)
	75+	56.7 ± 1.2 (1795)	49.7 ± 2.1 (586) *
	18-Mo		
	All	36.6 ± 0.8 (3996)	32.1 ± 1.9 (636) *
	20-44	44.2 ± 3.8 (176)	26.4 ± 10.1 (19)
	45-54	39.9 ± 1.9 (642)	39.8 ± 6.7 (53)
	55-64	41.3 ± 1.6 (1032)	48.0 ± 4.7 (115)
	65-74	36.2 ± 1.4 (1219)	37.3 ± 3.9 (165)
	75+	27.7 ± 1.6 (927)	21.3 ± 2.6 (284) *
04-13	6-Mo RSR		
	All	71.3 ± 0.4 (15619)	63.9 ± 1.0 (2492) ***
	20-44	77.7 ± 1.8 (549)	57.4 ± 5.9 (73) **
	45-54	75.3 ± 0.9 (2277)	74.4 ± 2.7 (273)
	55-64	75.2 ± 0.6 (4908)	73.8 ± 1.9 (561)
	65-74	73.7 ± 0.7 (4345)	70.9 ± 1.9 (601)
	75+	59.2 ± 0.9 (3540)	51.5 ± 1.7 (984) ***
	18-Mo		
	All	43.1 ± 0.7 (5730)	40.4 ± 1.7 (940)
	20-44	47.1 ± 3.7 (190)	27.3 ± 10.0 (21)
	45-54	43.7 ± 1.8 (795)	47.3 ± 4.6 (120)
	55-64	46.6 ± 1.2 (1850)	48.6 ± 3.4 (220)
	65-74	46.7 ± 1.3 (1563)	49.8 ± 3.4 (234)
	75+	32.7 ± 1.4 (1332)	26.6 ± 2.6 (345) *

Data are means ± standard error of the mean, with number of patients in parentheses. Abbreviations: Mo, month; RSR, relative survival rate; SEM, standard error of the mean.*$p < 0.05$, **$p < 0.001$, and ***$p < 0.0001$ for comparisons with the Male group.

Table S4. **12-month relative survival rates of EAC patients according to race, age and three decades.**

Decade	Age Group	Race		
		White	Black	Other
84-93	12-Mo RSR			
	All	39.3 ± 1.0 (2462)	36.2 ± 5.9 (68)	37.9 ± 6.7 (54) ***
	20-44	37.3 ± 5.0 (94)	33.5 ± 15.8 (9)	50.0 ± 35.4 (2)
	45-54	49.9 ± 3.0 (289)	35.7 ± 1.7 (17)	40.1 ± 22.0 (5)
	55-64	43.6 ± 2.0 (665)	53.6 ± 1.7 (19)	46.9 ± 12.9 (15)
	65-74	36.1 ± 1.7 (822)	20.7 ± 0.7 (15)	27.1 ± 11.6 (15)
	75+	33.9 ± 2.1 (592)	25.7 ± 15.7 (8)	37.2 ± 12.2 (17)
94-03	12-Mo RSR			
	All	46.1 ± 0.5 (8728)	35.6 ± 3.1 (259) ***	43.8 ± 3.5 (209)
	20-44	52.7 ± 2.6 (381)	43.1 ± 13.3 (14)	50.1 ± 13.4 (14)
	45-54	50.4 ± 0.4 (1319)	37.7 ± 6.8 (51)	53.7 ± 9.5 (28)
	55-64	51.6 ± 0.1 (2188)	40.8 ± 6.0 (71)	43.2 ± 6.7 (57)
	65-74	47.4 ± 1.0 (2551)	30.1 ± 5.4 (76) *	42.9 ± 6.3 (65)
	75+	35.6 ± 1.1 (2289)	32.1 ± 7.2 (47)	37.6 ± 7.5 (45)
04-13	12-Mo RSR			
	All	51.0 ± 0.4 (17165)	45.6 ± 2.4 (489) *	48.6 ± 2.4 (457)
	20-44	56.1 ± 2.2 (575)	40.7 ± 11.6 (19)	41.6 ± 9.9 (28)
	45-54	55.1 ± 1.1 (2360)	45.6 ±4.8 (120)	58.5 ± 6.1 (70)
	55-64	56.3 ± 0.7 (5165)	50.7 ±4.1 (166)	47.6 ± 4.5 (138) *
	65-74	53.7 ± 0.8 (4735)	47.7 ±5.1 (110)	49.2 ± 5.2 (101)
	75+	38.5 ± 0.8 (4330)	32.4 ±5.9 (74)	44.9 ± 4.8 (120)

Data are means ± standard error of the mean and the number of patients in parentheses Abbreviations: Mo, month; RSR, relative survival rate; SEM, standard error of the mean.*$p < 0.05$, **$p < 0.001$, and **$p < 0.0001$ for comparisons with the White group.

Table S5. **6-month and 18-month relative survival rates of EAC patients according to race, age and three decades.**

Decade	Age Group	Race		
		White	Black	Other
84-93	6-Mo RSR			
	all	63.3 ± 1.0 (2462)	59.6 ± 6.0 (68)	63.8 ± 6.7 (54)
	20-44	67.1 ± 4.9 (94)	55.7 ± 16.6(9) *	100.0 ± 0.0 (2)
	45-54	77.3 ± 2.5 (289)	88.7 ± 7.9 (17)	80.0 ± 17.9 (5)
	55-64	68.8 ± 1.8 (665)	74.7 ± 10.2 (19)	60.2 ± 12.7 (15)
	65-74	59.0 ± 1.7 (822)	27.2 ± 11.7 (15)	53.8 ± 13.0 (15)
	75+	55.6 ± 2.1 (592)	25.7 ± 15.7 (8)	66.1 ± 11.8 (17)
	18-Mo RSR			
	All	26.7 ± 0.9	21.4 ± 5.1	28.7 ± 6.3
	20-44	26.7 ± 4.6	22.4 ± 13.9	50.0 ± 35.4
	45-54	33.3 ± 2.8	11.9 ± 7.9	40.1 ± 22.0
	55-64	30.8 ± 1.8	38.5 ± 1.6	26.9 ± 11.5
	65-74	24.0 ± 1.5	14.0 ± 9.2	27.1 ± 11.6
	75+	22.2 ± 1.9	13.8 ± 12.9	25.3 ± 11.1
94-03	6-Mo RSR			
	all	66.3 ± 0.5 (8728)	56.4 ± 3.1 (259) **	66.4 ± 3.3 (209)
	20-44	73.5 ± 2.3 (381)	78.7 ± 11.0 (14)	85.7 ± 9.4 (14)
	45-54	71.9 ± 1.2 (1319)	61.1 ± 6.9 (51)	68.0 ± 8.8 (28)
	55-64	72.4 ± 1.0 (2188)	60.6 ± 5.9 (71)	62.8 ± 6.5 (57)
	65-74	67.0 ± 1.0 (2551)	52.3 ± 5.8 (76)	67.8 ± 5.9 (65)
	75+	55.1 ± 1.1 (2289)	44.4 ± 7.5 (47)	61.8 ± 7.5 (45)
	18-Mo RSR			
	All	35.3 ± 0.5	21.6 ± 2.6 **	29.8 ± 3.2
	20-44	39.7 ± 2.5	21.6 ± 11.0	28.6 ± 12.1
	45-54	38.7 ± 1.4	27.9 ± 6.4	32.3 ± 8.9
	55-64	39.9 ± 1.1	28.0 ± 5.5	34.4 ± 6.4
	65-74	36.9 ± 1.0	12.6 ± 3.9 ***	30.5 ± 5.9
	75+	26.2 ± 1.0	19.4 ± 6.2	21.8 ± 6.5
04-13	6-Mo RSR			
	all	70.5 ± 0.4 (17165)	61.8 ± 2.3 (489) **	70.2 ± 2.2 (457) *
	20-44	76.5 ± 1.8 (575)	52.3 ± 11.6 (19)	66.8 ± 9.1 (28)
	45-54	75.6 ± 0.9 (2360)	64.1 ± 4.5 (120) *	81.3 ± 4.7 (70)
	55-64	75.3 ± 0.6 (5165)	66.7 ± 3.7 (166)	73.3 ± 3.9 (138)
	65-74	73.7 ± 0.7 (4735)	63.9 ± 4.7 (110)	69.1 ± 4.7 (101)
	75+	57.6 ± 0.8 (4330)	46.7 ± 6.1 (74)	61.9 ± 4.6 (120)
	18-Mo RSR			
	All	40.3 ± 0.4	37.2 ± 2.4	37.4 ± 2.4
	20-44	42.8 ± 2.2	25.5 ± 11.3	32.4 ± 9.6
	45-54	43.1 ± 1.1	38.7 ± 4.8	42.4 ± 6.2

55-64	44.9 ± 0.7	44.3 ± 4.2	39.0 ± 4.4
65-74	43.6 ± 0.8	34.2 ± 5.0	42.6 ± 5.2
75+	29.2 ± 0.8	24.8 ± 5.6	29.2 ±4.5

Data are means ± standard error of the mean, with number of patients in parentheses. Abbreviations: Mo, month; RSR, relative survival rate; SEM, standard error of the mean. *$p < 0.05$, **$p < 0.001$, and ***$p < 0.0001$ for comparisons with the White group.

Table S6. **12-month relative survival rates of EAC patients according to SES, age and three decades.**

Decade	Age Group	SES	
		Low Poverty	Med-High Poverty
84-93	12-Mo RSR		
	all	39.5±1.3(1514)	38.8±1.5(1070)
	20-44	32.2±6.3(56)	43.0±7.1(49)
	45-54	50.6±3.6(192)	46.5±4.6 (119)
	55-64	45.9±2.5(403)	41.4±2.9(296)
	65-74	34.4±2.2(495)	37.3±2.6(375)
	75+	34.4±2.6(368)	33.0±3.2(249)
94-03	12-Mo RSR		
	All	48.7 ± 0.8 (4141)	43.4 ± 0.7 (5055) ***
	20-44	52.5 ± 3.5 (202)	52.1 ± 3.5 (207)
	45-54	55.9 ± 2.0 (620)	45.3 ± 1.8 (778) ***
	55-64	54.2 ± 1.6 (1047)	48.5 ± 1.4 (1269) *
	65-74	49.9 ± 1.5 (1184)	44.5 ± 1.3 (1508) *
	75+	37.0 ± 1.6 (1088)	34.3 ± 1.4 (1293)
04-13	12-Mo RSR		
	All	54.3 ± 0.6 (7379)	48.4 ± 0.5 (10732) ***
	20-44	58.5 ± 3.2 (245)	52.7 ± 2.7 (377)
	45-54	59.8 ± 1.6 (1030)	51.4 ± 1.3 (1520) ***
	55-64	59.7 ± 1.1 (2194)	53.4 ± 0.9 (3275) ***
	65-74	57.7 ± 1.2 (2015)	50.6 ± 1.0 (2931) ***
	75+	40.7 ± 1.2 (1895)	37.1 ± 1.0 (2629) *

Data are means ± standard error of the mean, with number of patients in parentheses. Abbreviations: Mo, month; RSR, relative survival rate; SEM, standard error of the mean. *p < 0.05, **p < 0.001, and ***p < 0.0001 for comparisons with the Low-Poverty group

Table S7. 6-month and 18-month relative survival rates of EAC patients according to SES, age and three decades.

Decade	Age Group	SES	
		Low Poverty	Med-High Poverty
84-93	6-Mo RSR		
	All	63.8 ± 1.3 (1514)	62.4 ± 1.5 (1070)
	20-44	62.6 ± 6.5 (56)	71.5 ±6.5 (49)
	45-54	77.2 ± 3.1 (192)	79.2 ±3.7 (119)
	55-64	70.3 ± 2.3 (403)	66.7 ±2.8 (296)
	65-74	58.7 ± 2.3 (495)	57.8 ±2.7 (357)
	75+	56.6 ± 2.7 (368)	53.9 ±3.3 (249)
	18-Mo RSR		
	All	26.2 ± 1.2 (1514)	27.0 ± 1.4 (1070)
	20-44	19.7 ± 5.3 (56)	34.8 ± 6.8 (49)
	45-54	33.3 ± 3.4 (192)	30.6 ± 4.3 (119)
	55-64	31.8 ± 2.4 (403)	29.9 ± 2.7 (296)
	65-74	22.5 ± 1.9 (495)	25.9 ± 2.4 (357)
	75+	22.4 ± 2.4 (368)	21.8 ± 2.8 (249)
94-03	6-Mo RSR		
	all	68.4 ± 0.7 (4141)	64.1 ± 0.7 (5055) ***
	20-44	74.3 ± 3.1 (202)	73.9 ± 3.1 (207)
	45-54	75.3 ± 1.7 (620)	68.3 ± 1.7 (778) *
	55-64	74.4 ± 1.4 (1047)	69.8 ± 1.3 (1269) *
	65-74	69.1 ± 1.4 (1184)	64.7 ± 1.3 (1508) *
	75+	56.7 ± 1.6 (1088)	53.6 ± 1.4 (1293)
	18-Mo RSR		
	All	37.3 ± 0.8 (4141)	32.8 ± 0.7 (5055) ***
	20-44	38.9 ± 3.5 (202)	38.5 ± 3.4 (207)
	45-54	41.9 ± 2.0 (620)	35.3 ± 1.7 (778) *
	55-64	42.7 ± 1.6 (1047)	36.8 ± 1.4 (1269) *
	65-74	38.9 ± 1.5 (1184)	33.8 ± 1.3 (1508) *
	75+	27.1 ± 1.5 (1088)	25.1 ± 1.3 (1293)
04-13	6-Mo RSR		
	all	73.1 ± 0.5 (7379)	68.3 ± 0.5 (10732) ***
	20-44	79.6 ± 2.6 (245)	72.5 ± 2.4 (377)
	45-54	79.5 ± 1.3 (1030)	72.3 ± 1.2 (1520) ***
	55-64	78.2 ± 0.9 (2194)	72.9 ± 0.8 (3275) ***
	65-74	76.7 ± 1.0 (2015)	71.1 ± 0.9 (2931) ***
	75+	59.0 ± 1.2 (1895)	56.4 ± 1.0 (2629)
	18-Mo RSR		
	All	43.6 ± 0.6 (7379)	37.8 ± 0.5 (10732) ***

20-44	45.0 ± 3.3 (245)	39.7 ± 2.7 (377)
45-54	47.8 ± 1.6 (1030)	39.6 ± 1.3 (1520) ***
55-64	48.3 ± 1.1 (2194)	42.4 ± 0.9 (3275) ***
65-74	48.2 ± 1.2 (2015)	40.1 ± 1.0 (2931) ***
75+	30.7 ± 1.2 (1895)	28.0 ± 1.0 (2629)

Data are means ± standard error of the mean, with number of patients in parentheses. Abbreviations: Mo, month; RSR, relative survival rate; SEM, standard error of the mean. *$p < 0.05$, **$p < 0.001$, and ***$p < 0.0001$ for comparisons with the Low Poverty group.

Table S8. Summary data for race distribution by SES and calendar period in patients with EAC during thirty years.

Decades	SES	Number	White	Black	Other
1984-2013					
	Total	28,098	80.06%	13.90%	6.05%
	Low Poverty	16,015	89.58%	6.23%	4.18%
	Med-High Poverty	12,083	67.43%	24.06%	8.52%
1984-1993					
	Total	7,965	73.95%	20.84%	5.21%
	Low Poverty	4,168	89.30%	9.21%	1.49%
	Med-High Poverty	3,797	57.10%	33.61%	9.30%
1994-2003					
	Total	9,373	80.36%	13.53%	6.11%
	Low Poverty	5,364	90.29%	6.15%	3.56%
	Med-High Poverty	4,009	67.07%	23.40%	9.53%
2004-2013					
	Total	10,760	84.31%	9.08%	6.61%
	Low Poverty	6,483	89.19%	4.38%	6.43%
	Med-High Poverty	4,277	76.92%	16.20%	6.87%
